# Surgery during pregnancy – results of a German questionnaire

**DOI:** 10.1515/iss-2020-0025

**Published:** 2020-10-08

**Authors:** Frauke Fritze-Büttner, Bettina Toth, Astrid Bühren, Katja Schlosser, Stefanie Schierholz, Beatrix Rumpel, Paul C. Helm, Ulrike M. M. Bauer, Maya Niethard, Sarah Prediger, Kristina Götzky, Joachim Jähne

**Affiliations:** Klinik für Allgemein- und Viszeralchirurgie, Sana Klinikum Lichtenberg, Berlin, Germany; Universitätsklinik für Gynäkologische Endokrinologie und Reproduktionsmedizin, Medizinische Universität Innsbruck, Innsbruck, Austria; Praxis für Psychosomatik und Psychotherapie, Honorary President of the German Association of Female Physicians, Murnau, Germany; Klinik für Allgemein-, Viszeral, Endokrine und Gefäßchirurgie, Agaplesion, Evangelisches Krankenhaus Mittelhessen, Giessen, Germany; Klinik für Chirurgie, Universitätsklinikum Schleswig-Holstein, Campus Lübeck, Lübeck, Germany; Kompetenznetz Angeborene Herzfehler e. V., Berlin, Germany; Klinik für Tumororthopädie, Helios Klinikum Berlin-Buch GmbH, Berlin, Germany; III. Med. Klinik, Sektion Ausbildungsforschung, Universitätsklinikum Hamburg-Eppendorf, Hamburg, Germany; Klinik für Allgemein- und Viszeralchirurgie, DIAKOVERE Henriettenstift, Hannover, Germany

**Keywords:** female surgeons, surgery during pregnancy, surgical career, work-life-balance

## Abstract

**Objectives:**

Worldwide, not only the number of female medical students, but also of female surgeons increases. Simultaneously, younger generations take a closer look to their work-life balance. With this in mind, it seems necessary to evaluate the expectations of female surgeons in particular with respect to pregnancy during their surgical career.

**Methods:**

Therefore, a nationwide survey was conducted in Germany from July to December 2016 under the auspices of the German Society of Surgery as well as the Professional Board of German Surgeons. The questionnaire involved 2,294 female surgeons and 1,843 complete records were evaluated.

**Results:**

Of the analyzed answers, 62% of the women (n=781) were operating during pregnancy. The joy of surgery (91.6%), followed by team spirit (57.1%), were the main motivations to perform operations while pregnant. Operative activity decreased from 30.8% in the first 3 months of pregnancy to 21.5% during the last three months. Regarding the possible complaints, e.g., leg edema, back pain, premature labor and vaginal bleeding, there were no significant differences between the women with or without activity in the operating room. Sick leave due to pregnancy (1–10 days) was stated by 40.4% of respondents.

**Conclusion:**

Despite strong legal regulations for pregnant surgeons, the survey showed that most female surgeons are eager to operate despite their pregnancy. The results also demonstrate no significant differences regarding complications during pregnancy- or pregnant-dependent absence from work. Hospitals and surgical departments are asked to establish proper working conditions for pregnant surgeons and pregnancy should not be an obstacle for a career in surgery.

## Introduction

Worldwide, the number of female students increases to almost 70% of all medical students. Simultaneously, the number of female surgeons shows a constant rise. Beside these gender-specific aspects, younger surgeons of the generation Y and the millennial tend to focus on an intact work-life balance, which represents a sharp contrast to the generation of the baby boomers. With this in mind, it seems necessary to evaluate the preferences of female surgeons how they could proceed with their surgical career in case of a pregnancy. This refers in particular to the attendance in the operating room.

Most likely, Germany has one of the strictest professional regulations for pregnant employees among the Western world, which meant in the past that pregnant surgeons were not allowed at all to work in the operating theater. The purpose is obvious: protection of the unborn child concerning infection and the risk of prematurity. In other countries like the United States most female surgeons work unmodified until delivery. There are several reports that being pregnant during surgical training time is associated with a negative stigma [1], [2]. As the duration of training is limited and allows no longer interruptions most publications try to answer the question if getting pregnant during residency makes sense at all. According to personal communications to one of the authors (K. Götzky), in Switzerland there is no uniform guideline, while in Austria similarly strict requirements as in Germany exist. In Norway, the hospitals decide for themselves, but there are general recommendations for pregnant women, e.g., no X-rays or exemption from night service in the 3rd trimenon. This leads to the present challenge: How can we manage the expectations of young female surgeons during pregnancy? It is possible to perform operations and continue the surgical training? In order to obtain more data of the situation and expectation of female surgeons during pregnancy a nationwide survey was performed and the data are presented herein.

## Methods

From July to December 2016 and under the auspices of the German Society of Surgery, the Professional Board of German Surgeons and the German Society of Orthopedics and Traumatology an online survey was conducted. In addition to sociodemographic data, the questionnaire included extensive information on the subject of “pregnancy and occupational activity”. The study involved 2,294 women working in surgery. Thousand eight hundred and forty three complete records were evaluated. Four hundred and fifty one were excluded because of incomplete information. Most of the participants came from general and visceral surgery. In relation to the size of the surgical subject, just as many colleagues from orthopedics and trauma surgery participated in the survey (see Table 1). The problem of wanting to operate and not be able to operate affects all surgical specialties, e.g., in particular, gynecologists, urologists and eye surgeons show a high level of interest in the subject, so that these fields of study were also included in the survey as were medical students. The demographic and professional data of the participants of the survey are listed in Table 1.

**Table 1:** Demographic data of the participants of the survey.

	n	%
**Participants**	1.843	
**Participants who were pregnant**	1.259	
Operating during pregnancy	781	62.0
Not operating during pregnancy	478	38.0
**Age at the time of survey, years**	1.843	
20–25	29	1.6
26–30	273	14.8
31–35	607	32.9
36–40	430	23.3
41–45	219	11.9
➢45	285	15.5
**Surgical specialty**	1.830	
Digestive surgery	501	27.4
Orthopedic and trauma surgery	320	17.5
General surgery	259	14.2
Reconstructive and plastic surgery	120	6.6
Gynecology	87	4.8
Pediatric surgery	77	4.2
Ophthalmology	77	4.2
Head and neck surgery	68	3.7
Neurosurgery	66	3.6
Urology	65	3.6
Vascular surgery	47	2.6
Heart surgery	31	1.7
Thoracic surgery	27	1.5
Various	85	4.6
**Current professional occupation**	1.841	
Intern	757	41.1
Resident	432	23.5
Senior resident	344	18.7
Chief resident	75	4.1
Head of a department	65	3.5
Surgeon in private practice	107	5.8
Medical student	48	2.6
Various	13	0.7
**Current employer**	1.818	
University hospital	386	21.2
3rd referral hospital	438	24.1
2nd referral hospital	377	20.7
Primary hospital	438	24.1
Private practice	160	8.8
Various	19	1.0

The statistical evaluation was carried out with the SPSS software (version 22). Descriptive methods were used as part of an exploratory data analysis. Since this is an adaptive questionnaire, the sample sizes may differ from the total number of participants, depending on the question to be evaluated. The percentages always refer to the group of participants who were asked the question or who answered the question. Due to the differing number of answers, the respective sample sizes are indicated for each question. Group differences were examined with the chi-square test.

## Results

Of the study participants 68.3% stated that they had been pregnant at least once during their employment. Of them 67.5% answered their own children’s question (no children: 7.6%, one child: 45%, two children: 36.3%, three children: 8.4%, more than three children: 2.7%). Almost 60% of the women had their first pregnancy during the internship, e.g., during the first 6 years of training.

**Figure 1:**
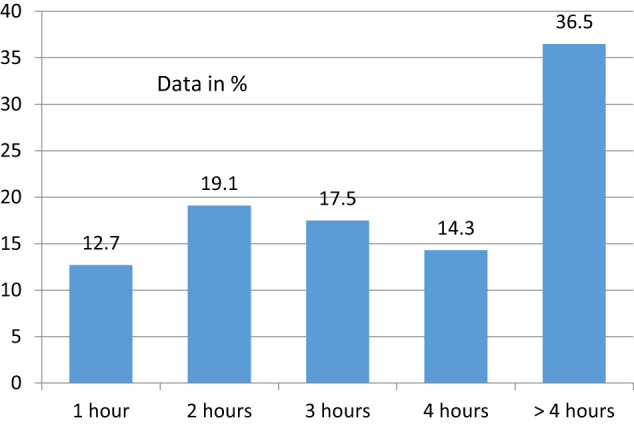
Hours in the operating room per day during pregnancy (n=640).

Sixty seven percent of women confirmed the question of surgical activity during pregnancy. These were asked about the factors influencing their decision regarding the operative activity. The joy of performing surgery had the greatest influence on the decision to continue operating during pregnancy (91.6%), followed by team spirit (57.1%). Only 15.9% stated that the head of the department expected them to do surgery. During the various stages of pregnancy, the operative activity dropped from 30.8% during the first 3 months of pregnancy to 21.5% within the last 3 months, but 25% of these surgeons spent more than 5 h in the operating room. Overall, 36.5% of all participants spent more than 4 h in the operating room (Figure 1).

**Figure 2:**
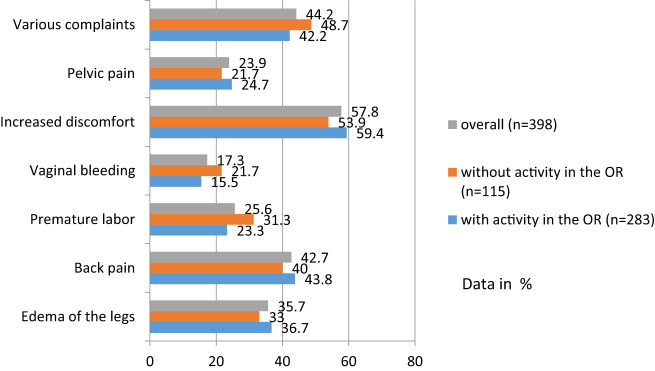
Physical complaints during the first pregnancy.

Of respondents 40.3% who were pregnant at least once said they had discomfort during pregnancy. Multiple answers were possible. The focus was primarily on increased malaise and back pain, followed by leg edema. A detailed overview of the physical symptoms during pregnancy, also depending on whether the colleagues had operated or not, are presented in Figure 2.

It was also asked what activities were allowed to perform during the period when they were not operating due to the pregnancy. In more than 60% of cases, pregnant surgeons were involved in patients’ and relatives talks as well as office works and patients’ reports. It appears that most pregnant surgeons are involved in administrative work, because only 17% worked in the emergency room and just 39% were taken blood samples, for example.

Seventy seven percent of respondents who had previously been pregnant answered the question of complications, with a quarter reporting complications. Among these complications, premature labor were most common, but still in only 9.7%. Early and late abortions occurred in 2, 8 and 0.8%, respectively. Only 5% of study participants saw a direct causal relationship between their complications during pregnancy and work. Almost 60% of the pregnant women gave birth within the 37th and 40th week of pregnancy and 28.5% later than the 40th week. Only 3.5% had childbirth before the 33rd week.

**Figure 3:**
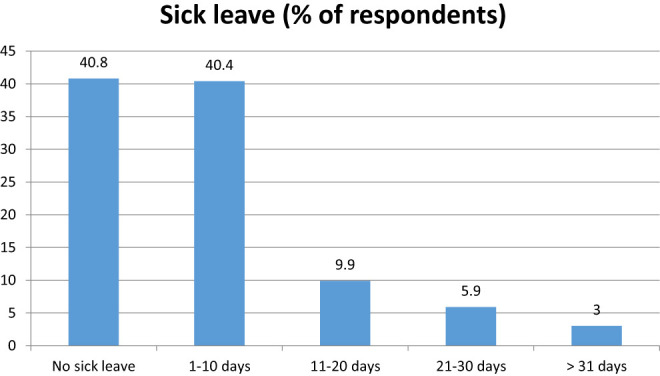
Sick days during pregnancy outside maternity leave (n=983).

Overall, 40.8% had no sick leave due to illness during the first pregnancy (Figure 3) and only 3% were 31 days or more absent due to illness.

Almost half of the study participants provided information of the time they informed the department director about their pregnancy. At first pregnancy, 64.3, 33 and 2.7% did it in the first, second and third trimester, respectively.

The colleagues who decided against surgery during pregnancy were asked about their motivations. In most cases—79.9 and 73.9%, respectively – cited “protection of the unborn life” as their main reason for stepping down from activity in the operating room.

Of all surgeons asked, 91% answered the question whether they would like to be in the operation room again during another pregnancy. It was found that both women who had previously been pregnant and women who were not yet pregnant would like to continue their operative activity during another pregnancy (Table 2).

**Table 2:** Information on the continuation of the operative activity during pregnancy.

Have you ever been pregnant during your professional career?	Would you (again) take over surgical activities in the operation room?	n	%
Yes	Yes	526	73.8
	Perhaps/Do not know	104	14.6
	No	83	11.6
No	Yes	745	77.2
	Perhaps/Do not know	154	16.0
	No	66	6.8

## Discussion

Not only the number of female medical students but also the number of female surgeons is steadily rising. Therefore, in the future, women will increasingly shape the personnel structure in the hospitals. This challenges chief physicians and directors of surgical departments to adapt the structure of the clinic and the working conditions to the changed expectations of the younger co-workers and in particular with respect to possible pregnancies of the female employees. However, it is undisputed that women in surgery often face considerable difficulties in coping with their dual role as doctors and mothers. For example, in a survey on the satisfaction of female surgeons in Germany, only 20% stated that work and private life were compatible. One-third of the respondents expressed significant problems [3].

In the current survey, female physicians in surgical training (41.1%) represent the largest group. This reflects the interest of females in continuing their education and also dealing with the topic of “surgery during pregnancy”. Already Welcker et al. stated that, in particular, continuing education was not considered an ideal time to have a child [4]. The reasons for this opinion were primarily the extension of the training period and the delay in the professional development due to the restrictive interpretation of the Maternity Protection Act. Nevertheless, in our study, 56.6% of female surgeons in the first pregnancy were employed as interns. A total of 67% of the participants also operated during their pregnancy. The most frequently mentioned motivation was the “joy of operating”. This is in line with the results of the survey by Leschber et al. in 2008: women who did their surgical work during pregnancy were more fulfilled in their professional situation. Thus, 61.4% of respondents stated that they were more satisfied with their occupational activity [3].

It is striking that in our study only two-thirds of pregnant women informed their chiefs within the first trimester, one-third between the second and third trimester and a small part even as late as in the last trimester. Unfortunately, due to the low number of cases and the questionnaire, which was often not completely answered, we were unable to make a statistical statement as to the extent to which the professional position had an influence on the time of the announcement of the pregnancy. Yet, Knieper et al. noted that with increasing responsibilities and leadership, the pregnancy tended to be communicated 5 weeks later, with a later withdrawal from surgery (18.4 vs. 24.7 weeks gestational age) [5].

One-third of those who had given full details of the number of hours per operation were in the operating room for 4 h or even more. It can be assumed that these surgical times were mainly in the beginning of pregnancy and the pregnancy had probably not yet been reported. As expected, the number of hours pregnant women operated decreased from the first to the last trimester. In total, nearly two-thirds of our study participants operated up to 4 h in the first trimester, about one-third for 5–6 h, and just under 10% for more than 7 h per day. By the third trimester, the ratio reversed in favor of shorter operating times. In the study by Knieper et al., the average number of hours in surgery in the first trimester was 4.0 ± 2.8 h and in the last trimester 1.1 ± 2.7 h [5].

The aim of the currently relevant German Maternity Protection Act is to “protect the physical and mental health of the woman and her child at work, education and study places during pregnancy, after childbirth and while breastfeeding”. On the one hand, this requires the pregnant woman to report pregnancy as early as possible, so that appropriate safety precautions can be taken. On the other hand, this also means that in addition to the infection protection, the use of stab-proof instruments and the type of anesthesia at the workplace “operating room” must be optimized and adapted to the pregnant woman.

Our survey showed that there were no significant differences in physical complaints between operating andnonoperating pregnant women. About 9.7% of respondents named premature labor when asked about pregnancy complications, and 5% had premature birth. Only 5% of the sample saw a direct connection between the complication during pregnancy and the professional activity. In the survey by Knieper et al., 7.1% suffered from premature births. Three of the respondents at that time saw a correlation with their operative activity [5]. The rate of prematurity (birth before the 37th week of pregnancy) has remained stable according to statistics in recent years. However, the number of extreme preterm births (<28 weeks of gestation) has increased by up to 65% [6]. It should be noted, however, that 25–30% of premature births are also iatrogenic due to a fetal growth restriction, preeclampsia, preexisting maternal diseases or multiple pregnancies [7]. The causal factors in these cases are risk factors in balanced socioeconomic conditions, in particular the increase in maternal age and the increasing prevalence of arterial hypertension and diabetes mellitus [8]. In comparison with other European countries, Germany has one of the highest premature birth rates, at 8.6%, with good chances of survival, especially for the smallest preterm infants <1,000 g [9].

In our survey regarding, the number of sick days outside of the general employment ban, it could be shown that more than 80% of the participants were missing from work for only 1–10 days due to illness during pregnancy. If one compares this figure with the statistical data of the Federal Ministry of Health with 12.9 average days lost in 2013, this indicates a high willingness to work during pregnancy [10].

Irrespective of the fact that the Maternity Protection Act provides this option to women doctors who wish to be active in surgery, it remains to be respected that pregnant female surgeons refuse activities in the operating room. In our study, 149 female colleagues said they did not want to be (again) active during another pregnancy. Two hundred and fifty eight were undecided. Only 135 colleagues gave further information on their motivation. The primary concern was the harm of the unborn life by the operative activity.

A total of 75.7% of the study participants stated that they were continuing their surgical activity during pregnancy. This high level of agreement coincides with the results of Knieper et al. Here it was even 88%, who could imagine an operative activity in a new pregnancy [5]. In our opinion, it is crucial that every woman should make her decision to operate in pregnancy voluntarily and this decision can be revoked at any time without any professional disadvantages. Due to the strong desire of female surgeons to continue their work in the operating room, the common goal of all surgeons and in particular the mainly male chiefs should be to create a safe workplace for pregnant female doctors.
