# Supplementary material for: Surgery during pregnancy – results of a German questionnaire
Source: Innov Surg Sci. 2020 Oct 8;5(1-2):21–6. doi: 10.1515/iss-2020-0025 (PMC7798309; doi:10.1515/iss-2020-0025)
Supplement: Supplementary file 1 [file iss-5-20200025-s001.pdf]

Reviewer Assessment

Frauke Fritze-Büttner et. al: Surgery During Pregnancy – Results of a German Questionnaire.

Comments by the Editor-in-Chief to Original Submission

The increasing number of young female surgeons in training and residency challenges all surgical clinics and departments with respect to possible pregnancies. Various nations have various legal regulations and professional guidelines to give advice what kind of surgical activity is allowed or may not be undertaken while pregnant. The paper presented by Fritze-Büttner and colleagues from Germany demonstrates on the basis of a nationwide survey that female surgeons in training are eager to be in the operation room to perform surgery. Therefore, hospitals should take precautions to make operations during pregnancy safe and feasible to meet the expectations of female surgeons in order to pursue a career in surgery. The topic appears to be of high impact so that the Editorial Office decided to dispense the double-blind peer review process. This seems to be justified due to the fact that the paper was already published in German after a peer-review.
